# Supplementary material for: DMRforPairs: identifying Differentially Methylated Regions between unique samples using array based methylation profiles
Source: BMC Bioinformatics. 2014 May 15;15:141. doi: 10.1186/1471-2105-15-141 (PMC4046028; doi:10.1186/1471-2105-15-141)
Supplement: Additional file 2 — DMRforPairs output for the comparison of A431-MCF7 and NA17018-NA17105. Please start from the HTML files in each folder. Available via the BMC Bioinformatics website. [file 1471-2105-15-141-S2.zip › 1394847754114233_MOESM2_ESM/A431_MCF7/figures/31610.pdf]

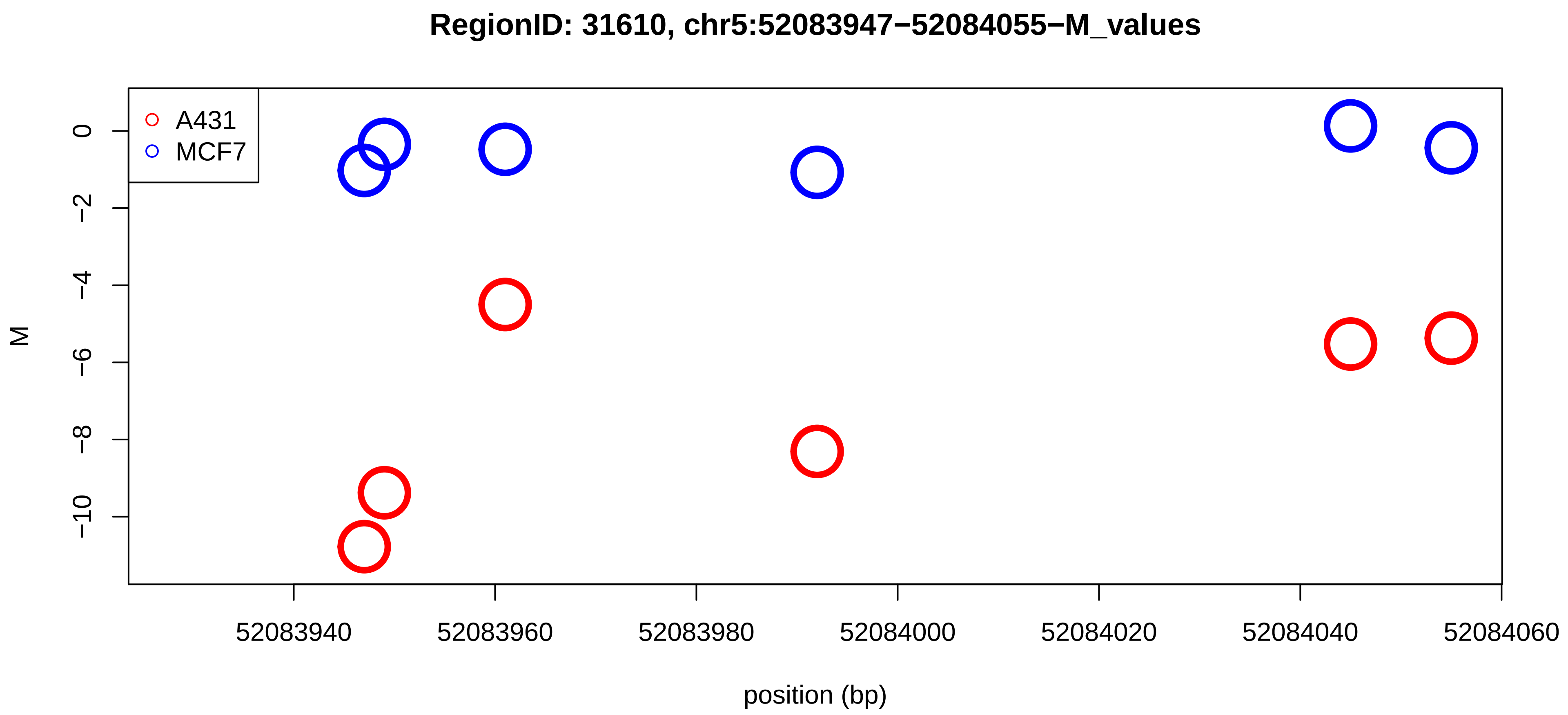
RegionID: 31610, chr5:52083947−52084055−M_values
A431
MCF7
M
0
−2
−4
−6
−8
−10
52083940
52083960
52083980
52084000
52084020
52084040
52084060
position (bp)

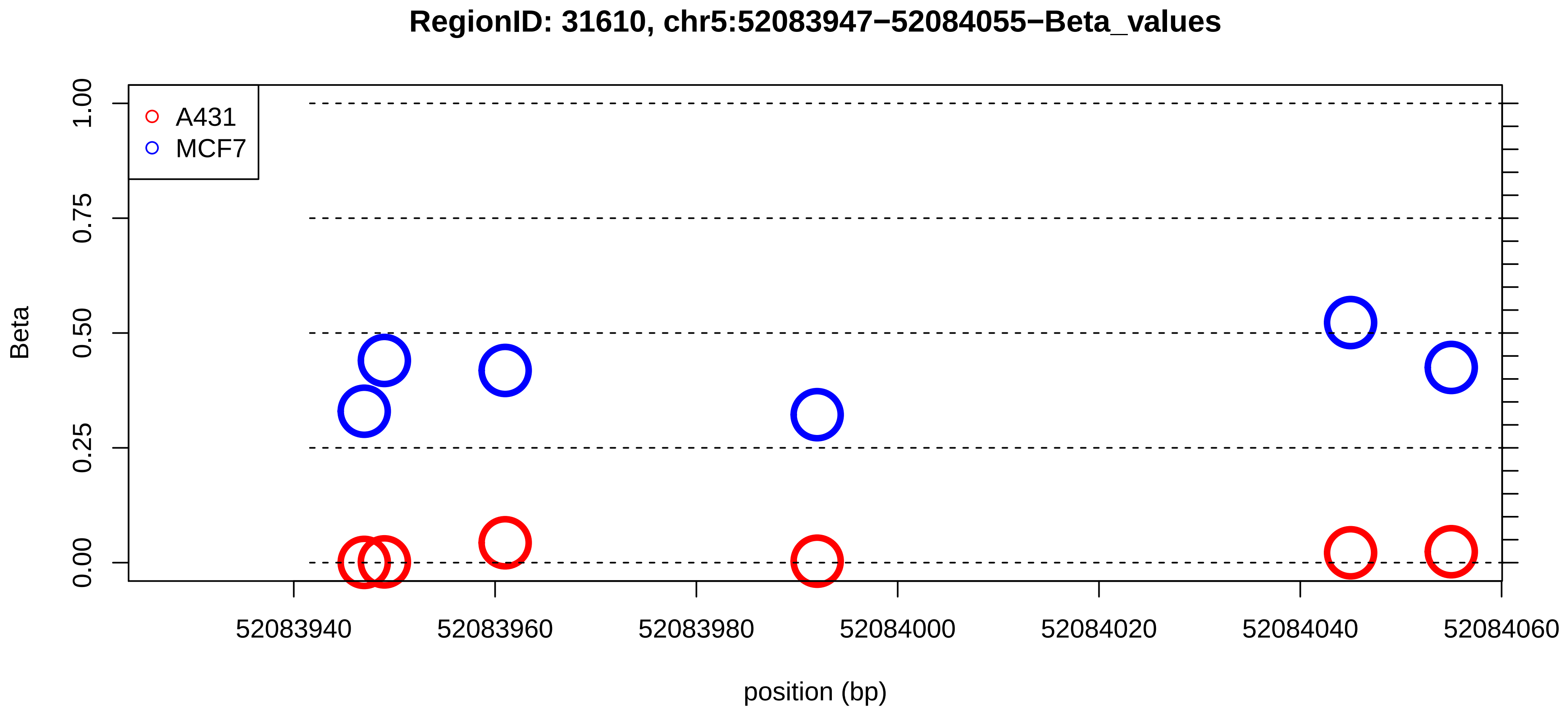
RegionID: 31610, chr5:52083947−52084055−Beta_values
A431
MCF7
Beta
0.00
0.25
0.50
0.75
1.00
52083940
52083960
52083980
52084000
52084020
52084040
52084060
position (bp)

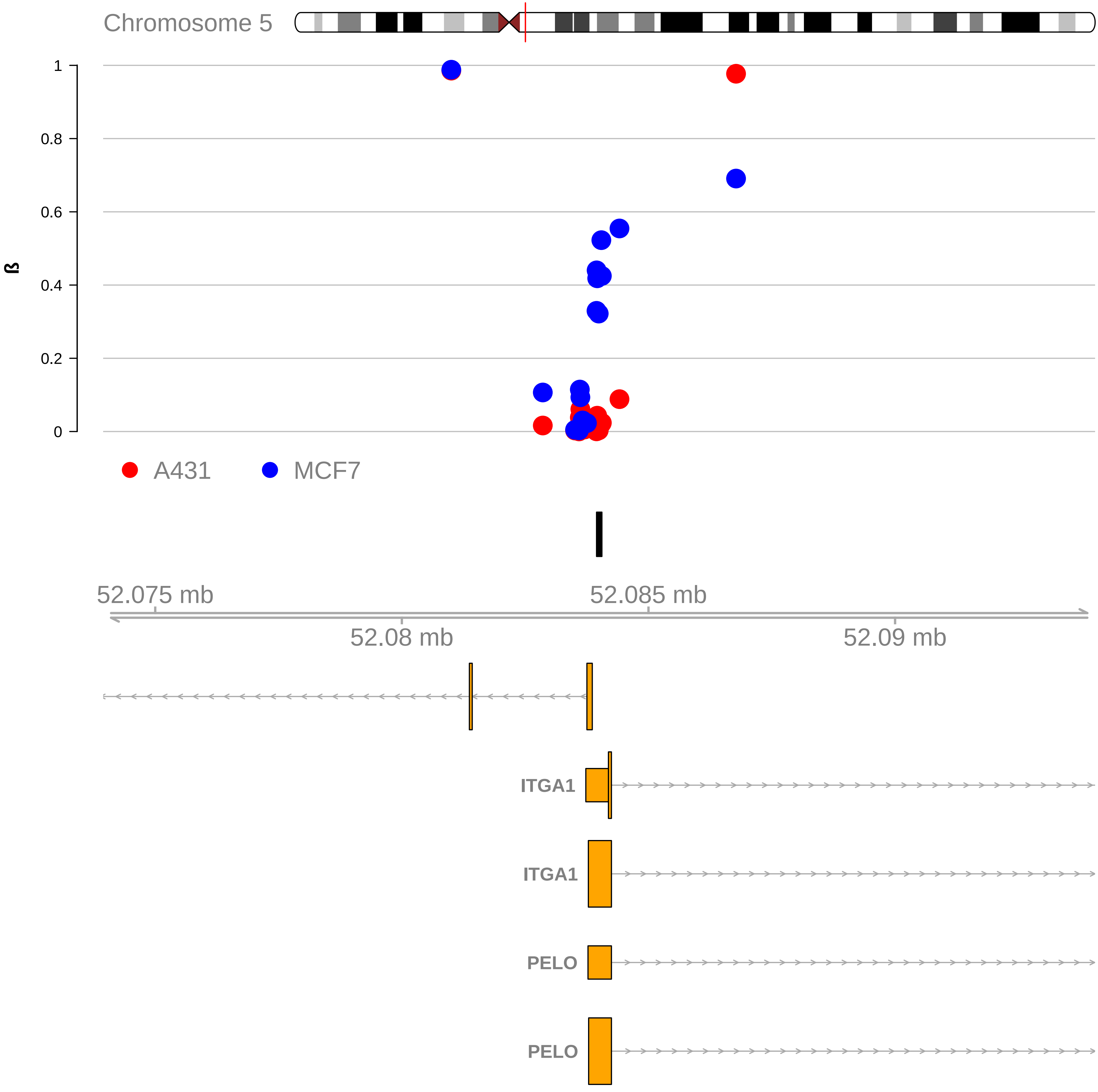

Chromosome 5
1
0.8
0.6
0.4
0.2
0
ß
A431
MCF7
52.075 mb
52.085 mb
52.08 mb
52.09 mb
ITGA1
ITGA1
PELO
PELO
